# Systems-biology dissection of eukaryotic cell growth

**DOI:** 10.1186/1741-7007-8-62

**Published:** 2010-05-24

**Authors:** Teresa M Przytycka, Justen Andrews

**Affiliations:** 1 National Center for Biotechnology Information, NLM, NIH, 8000 Rockville Pike, Bethesda, MD 20814, USA; 2 Department of Biology, Indiana University, 915 E Third Street, Bloomington, IN 47405, USA

## Abstract

A recent article in *BMC Biology *illustrates the use of a systems-biology approach to integrate data across the transcriptome, proteome and metabolome of budding yeast in order to dissect the relationship between nutrient conditions and cell growth.

See research article http://jbiol.com/content/6/2/4 and http://www.biomedcentral.com/1741-7007/8/68

## Commentary

It is generally appreciated that organismal phenotype is a function of both the genotype and the environment. However, most recent studies have focused on understanding the relationship between genotype and phenotype. Indeed, genetic variations are easier to quantify, data are abundant, and new methods continue to emerge. Utilizing genomic-scale gene expression and various types of molecular interaction data, several groups have started to address the challenge of identifying the molecular pathways that underlie the translation of different genotypic perturbations into corresponding phenotypic output, for example, a particular disease. In contrast, little has been done to dissect the relationship between the environment and the phenotype at the systems-biology level.

Understating the relationship between an environmental factor and a phenotype involves uncovering biomolecular pathways participating in a given environment-phenotype response. Just as various genotypic variations might lead to the same disease, various environmental perturbations often lead to the same phenotypic response. In such a case it is to be expected that the responses to these signals involve common pathways, which in turn begs several questions. What are they? What are the intermediate steps before the signals converge to such a common pathway? Which pathway is signal specific? Which molecules are involved and what is the crosstalk between different response pathways? Finally, and most important, where do we start tackling this complex problem?

Several groups have begun applying systems-level approaches to study the mechanisms that underlie cellular responses to changing environmental conditions, and these studies suggest that we are on the right path. For example, DeRisi *et al*. [1] investigated the gene-expression response accompanying the metabolic shift from fermentation to respiration in the yeast *Saccharomyces cerevisiae*. In a contrasting model-based approach, Herrgard *et al*. [2] used a reconstructed nutrient-controlled transcriptional regulatory network, and coupled it with a genome-scale metabolic network to predict growth phenotypes of transcription factor knockout strains. Moxley *et al*. [3] developed a model-based approach to correlate mRNA and metabolic flux data. Yet another approach was taken by Bradley *et al*. [4], who measured and analyzed the metabolomic and transcriptional responses of *S. cerevisiae *to carbon and nitrogen starvation. To uncover functional relations between genes and metabolites, they developed an approach based on Bayesian integration of the joint metabolomic and transcriptomic data. These and related studies helped to illuminate several aspects of molecular and/or network-level responses to a changing environment. However, as in the case of genotype-phenotype relationships, we would also like to measure and explain the dependencies between environment and higher-level phenotypes, such as the relationship between nutrients and growth.

The relationship between a cell's nutritional resources (environment) and its growth rate (phenotype), is complicated by the fact that cells affect their own environment by consuming nutrients. This problem can be circumvented by utilizing a chemostat - a device that simultaneously controls the amount of nutrients, cell population size and waste products to clamp the environment [5]. This is achieved by continuously supplying nutrients and, at the same rate, removing the culture. The level and rate of supply of a selected nutrient, the so-called limiting nutrient, is used to control the cell growth rate. For a given flux (growth rate), the steady state is achieved by (self) balancing the population size and nutrient concentration within the device. This provides a setting for studying the impact of the equilibrium nutrient concentration (corresponding to a given growth rate) on transcriptome, proteome and any other component that can be systematically measured. In this issue of *BMC Biology*, Steven Oliver and his colleagues (Gutteridge *et al*. [6]) extend the analysis of data from an earlier study by the same group using the chemostat setup [7] to focus on the effects of growth where different nutrients are limiting. A similar approach has been used by Boer *et al*. [8]. The data are analyzed along two distinct axes - a multivariate analysis of growth conditions (Nutrient availability × Growth rate), and an integration of data across three 'omes'.

## From nutrient supply to growth rate

**Figure 1:**
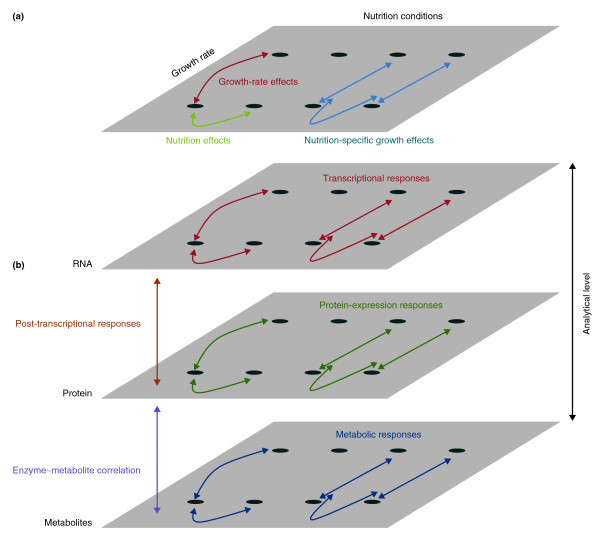
**Analytical approaches**. **(a) **Multivariate analysis of growth rate and nutrition conditions. Comparing cellular responses to varying growth rates and nutrition conditions allows the dissection of growth-rate effects (red), nutrition effects (green) and nutrition-specific growth effects (blue). **(b) **Systems-biology analysis of cellular responses. Assaying cell responses at the transcript, protein, and metabolite levels allows the analysis of transcriptional (red), protein expression (green), and metabolic (blue) responses. Comparing the transcription and protein-expression responses allows the inference of post-transcriptional responses (orange). Comparing the protein expression and metabolic responses allows the inference of enzyme-metabolite correlations (purple).

The multivariate analysis examines the response of yeast cells to different nutrition conditions and over differing growth rates. Cells were cultured in media limiting for either glucose, ammonium, phosphate, or sulfate; while the growth rates were set at doubling times of either 3.5, 7 or 10 hours. This allowed the effects of nutrition to be disentangled from secondary effects associated with altered growth rates (Figure 1a). Effects that were only associated with growth rate were identified as variation that was common to the different growth rate conditions across all limiting nutrients (red in Figure 1a). Effects that were only associated with different nutrient conditions were identified as variation that was specific to a nutrition treatment averaged across different growth rates (green in Figure 1a). Finally, nutrition-specific growth-rate effects were identified as growth-rate effects that were found in specific nutrient conditions (blue in Figure 1a).

The integrative systems biology analysis involved examining the cellular responses at the transcriptomic, proteomic and metabolomic levels (Figure 1b). The transcriptomic responses were assayed using microarrays, the proteomic responses were assayed using isotope tags for multiplexed relative and absolute quantification (iTRAQ) and the metabolomic responses were assayed using gas chromatography coupled to time-of-flight mass spectrometry (GC/TOF-MS). Comparing responses at the transcriptomic and proteomic levels allows the inference of post-transcriptional regulatory effects (orange in Figure 1b). For instance, post-transcriptional regulation can be inferred for a gene if the protein-level response to a treatment differs markedly from the transcriptional response; for instance, a marked protein response in the absence of a transcriptional response. Finally, comparing the responses of specific metabolites to the responses of the proteins involved in their metabolism allows correlations between metabolites and cognate enzymes to be explored (magenta in Figure 1b).

In an earlier study Oliver's group identified a response to altered growth rates that was common across nutritional conditions [7]. The current study [6] examines the nutrition-specific effects, and the nutrient and growth-rate-dependent effects. The analysis of nutrient-specific effects revealed that the cells have distinct responses to limitations of each nutrient, of which the response to carbon (glucose) limitation is by far the most dramatic. At the transcriptional level, around 1,200 genes were up- or downregulated under limiting carbon compared with around 100 to 200 for the other three nutrients. In addition, the Gene Ontology (GO) term annotations of transcripts and proteins responding to carbon limitation are largely distinct from those responding to limitation of the other nutrients. The analysis of growth-rate-dependent effects in each nutrition condition revealed a more robust response, with both a greater number of genes involved (around 1,400 to 3,300 across all nutritional conditions) and a greater range of responses at the transcriptional and protein levels. In this case, the GO annotations of the responding transcripts and proteins were similar across all four nutrition conditions and prominent functions included ribosome- and translation-related functions. Again, only a handful of genes were found to be outliers in terms of proteome/transcriptome comparisons.

The integration of transcriptomic, proteomic and metabolomic data provides a more systems-wide view of the cell state than one type of data can. Although all the assays aimed at being as comprehensive as possible, only the transcriptomic data approach the system-wide level. The micoarrays detected transcripts from 6,084 protein-coding genes, whereas the iTRAQ proteomic data detected peptides corresponding to 1,870 open reading frames (ORFs), and the metabolomic data are restricted to a few hundred metabolites (around 400 metabolites were detected and around 100 unambiguously identified and quantified). Nevertheless, these studies provide insights otherwise not possible when one is limed to one slice of the cell's 'omes'.

By comparing the transcriptional responses to the changes in proteins, Gutteridge *et al*. [6] were able to infer post-transcriptional effects. They found that the overall correlation between transcriptional and protein expression responses was low, and suggest that this reflects pervasive post-transcriptional regulation. Nevertheless, they identified relatively few genes that met their criteria of notable outliers in the proteome/transcriptome comparisons. For instance, across the nutrition conditions only 11 genes were notable outliers. These included cases of positive- and negative-post-transcriptional control, although the mechanism(s) are as yet unknown. Similarly, correlating changes in metabolites with the enzymes that catalyze their production or consumption allowed inferences regarding metabolic responses. Here the data fall short of the hope for a systems-level picture of the cell's behavior. In most cases there was little correlation between the levels of enzymes and the corresponding metabolites. The authors suggest that this reflects the fact that metabolite levels are controlled by systems-level properties of metabolic pathways, which is reasonable given that it has long been known that metabolite levels are well buffered against changes in enzyme concentrations [9]. Given the sparseness of the metabolomic data, and to a lesser extent the proteomic data, a fuller picture must probably await further technological advances.

This is a rich dataset. The analyses to date have largely focused on a high-level analysis of groups of genes with common GO annotations. This revealed that limiting each of the four nutrients tended to induce responses that were moderate in range, but distinct across the nutritional conditions, with carbon limitation producing a unique and dramatic response. On the other hand, the nutrient- and growth-rate-dependent analysis revealed a wider range of transcriptomic and proteomic effects, but which were qualitatively similar across the nutrient conditions. While this analysis naturally focused on genes of known function as a means to biological interpretation, further mining of the data is likely to be fruitful. For instance, Oliver and colleagues noted in their earlier paper [7] that a significant number of genes that were downregulated at increased growth rate are of unknown function. Can these and related data be used to infer the possible functions of these genes?

## Looking ahead

**Figure 2:**
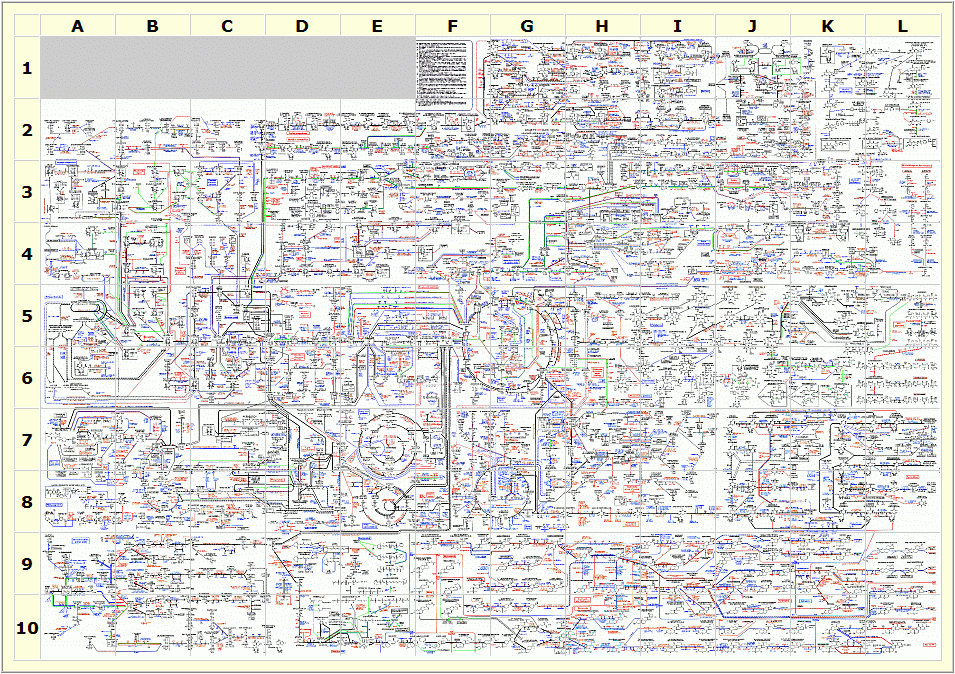
**Biochemical pathways**. Graphical summary of the metabolic pathways found in prokaryotic and eukaryotic cells. Notes and definitions of abbreviations can be found at the ExPASy proteomics server [10]. Reproduced with permission of Roche Applied Science, ^© ^1993 Boehringer Mannheim GmbH - Biochemica.

Periods of rapid scientific progress are often marked by the coming together of previously distinct fields into a synthesis. In the 20th century we saw the union of genetics and evolutionary biology, genetics and molecular biology, and molecular and developmental biology. Genomics has been widely seen as a key component of future advances that take advantage of complete and information-rich data, and the gathering of these datasets has become more and more common. While a full systems-biology view remains still more promise than reality, one can imagine that some of the more important avenues of exploration will involve the integration of datasets related to processes that we know quite a bit about. We know quite a lot about the cell cycle through, for example, screens for temperature-sensitive lethals in yeast and through the genome-wide analysis of gene expression during the cell cycle. We also know quite a lot about primary metabolism though the combined efforts of biochemists in the last century, and how enzyme kinetics is translated into flux through metabolic networks (Figure 2).

The new study by Oliver and colleagues [6] is beginning to expanding the dimentionality of this map, and is significant at two levels. First, it pioneers an integrative systems-biology approach, where cellular responses are simultaneously analyzed at the transcriptomic, proteomic and metabolomic levels. Second, it contributes to efforts leading to a comprehensive view of the many ways in which a eukaryotic cell alters its state in response to external conditions. The current work uncovers specific dependencies and responses. Much still needs to be done to put these relationships into the context of networks, pathways and predictive models. The integrated systems biology of metabolism is likely to be a very important part of the synthesis of the information deployed by the genome, the enzymes that do the work, and the substrates and products that enzymes act upon and produce.
